# Exploring Neural Networks and Related Visualization Techniques in Gene Expression Data

**DOI:** 10.3389/fgene.2020.00402

**Published:** 2020-05-15

**Authors:** Roni Wilentzik Müller, Irit Gat-Viks

**Affiliations:** School of Molecular Cell Biology & Biotechnology, Tel Aviv University, Tel Aviv, Israel

**Keywords:** neural networks, saliency maps, activation maximization, multiclass classification, deep learning, structured data, gene expression, biological traits

## Abstract

Over the past decade, neural networks have become one of the cutting-edge methods in various research fields, outshining specifically in complex classification problems. In this paper, we propose two main contributions: first, we conduct a methodological study of neural network modeling for classifying biological traits based on structured gene expression data. Then, we suggest an innovative approach for utilizing deep learning visualization techniques in order to reveal the specific genes important for the correct classification of each trait within the trained models. Our data suggests that this approach have great potential for becoming a standard feature importance tool used in complex medical research problems, and that it can further be generalized to various structured data classification problems outside the biological domain.

## Introduction

With the rapid rising of deep learning research over the past years (Lecun et al., 2015), neural networks have recently become one of the key models in computational biology, prominent in fields such as medical diagnosis, medical genomics, regulatory genomics, and cellular imaging; to name but a few (Angermueller et al., 2016; Leung et al., 2016; Min et al., 2016; Jones et al., 2017; Eraslan et al., 2019). Within these domains, artificial neural networks have been shown to encompass great potential in learning complex relationships from high-throughput omics data such as genomics, proteomics, metabolomics and alike (Grapov and Fahrmann, 2018; Zhang et al., 2019). Lately, classification problems, one of the most popular domains in deep learning (Lecun et al., 2015), gained focus in medical analysis by studies where molecular data have been suggested for classification of biological or medical traits (Chen et al., 2014; Dwivedi, 2018; Kong and Yu, 2018). In this study we methodologically explore the use of neural networks for classifying biological traits based on gene expression levels, and strive for the identification of trait-specific genes that are important for successful classification. For this purpose, we utilize a dataset of expression levels of immunological genes measured in healthy individuals in response to extracellular stimulations (Lee et al., 2014). We test three biological traits: the *gender* and the *ethnicity* of the individual from whom the immune cells were derived, along with the extracellular *stimulation* following which the expression levels were measured. Formally, each biological trait corresponds to a multiclass classification problem according to the number of distinct classes related to this trait (for example, the four classes within the ethnicity trait are “African-American,” “Caucasian,” “East-Asian,” and “Multi-racial”).

Naturally, the latest turmoil of deep learning studies has also brought attention to studies that focus on basic questions regarding the application of neural networks; whether neural networks modeling improves on gold-standard methods, whether it worth the cost of adding complexity to model interpretability, or how an appropriate network architecture should be chosen (Montesinos-López et al., 2018; Traits et al., 2018; Yu et al., 2019). As our first contribution in this paper, we provide a methodological study for examining the benefits of neural network modeling for the tested biological traits classification based on gene expression data and discuss different possibilities for network architectures.

The second contribution of this paper relates to the estimation of feature importance through deep learning visualization techniques. Here we aim to shed light on patterns within the input that are important for the prediction of a given model. Particularly, for each of the biological traits we strive to identify the specific genes important for its classification. We demonstrate how feature importance analysis can be conducted using two visualization techniques commonly used in computer vision. We start by utilizing Saliency Maps (Simonyan et al., 2013) to address the challenge of highlighting input features essential for the correct classification of a given sample. Then, we turn to utilizing Activation Maximization (Erhan et al., 2009) to address the challenge of uncovering features that have strong impact on the model prediction for each class. Notably, we exploit the fact that the gene expression input is a structured numerical data – we take advantage of its tabular format by averaging per gene across all samples in a class – to show that the two visualization techniques converge to similar results. Finally, we examine the specific genes that obtained the highest importance estimations for each of the tested biological traits and find solid biological reasoning for why these particular genes are relevant for the classification process of a trait. Various feature importance methods have been proposed along the years (Eraslan et al., 2019), however, to the best of our knowledge, this is the first time deep learning visualization techniques are used to estimate feature importance of structured numerical data, specifically for classifying biological traits. It is also the first demonstration that the two visualization techniques converge to similar results when applied on structured numerical data. As complex neural networks nowadays become a major part of biological modeling, we believe the suggested techniques may be an important addition to our arsenal of cutting-edge model-interpretability techniques.

## Results

### Investigating Neural Network Architectures for Classifying Biological Traits

**FIGURE 1:**
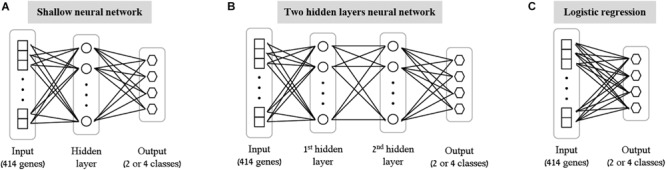
Tested neural network architectures. Presented are the three architectures examined in this paper: shallow neural networks with one hidden layer **(A)** or two hidden layers **(B)** of varying sizes, and a neural network without hidden layers, equivalent to multiclass logistic regression **(C)**. In all cases the input layer is an expression level panel of 414 genes, while the output layer consists of one neuron for each class of the biological trait (either 2 neurons for gender classification, or 4 neurons for stimulation and ethnicity classification). Different number of neurons (ranging between 2 and 64) were tested for the hidden layers within the shallow and the two hidden layers architectures.

As the first step of our work, we decided to focus on some basic questions regarding neural networks – whether we can use them as a simple tool for classifying biological traits, what is the level of accuracy that can be obtained, and which architectures should be used for this purpose. We have utilized a previously published dataset (Lee et al., 2014) of 2441 samples, each is a panel of 414 immune-related genes whose expression levels were measured in healthy individuals (section “Gene Expression Data and the Classified Biological Traits”). We chose to examine three categorical biological traits: (i) the extracellular stimulation following which the expression levels of the genes were measured (LPS, dNS1, IFNβ or no stimulation), (ii) the gender of the individual from which the immune cells were derived (male or female), and (iii) the ethnicity of the individual (African-American, Caucasian, East-Asian or Multi-racial). For each of these three biological traits we explored various neural network architectures of multiclass classification models (Figure 1 and section “Neural Network Architectures”). In all cases, the input layer of the classifier is an expression panel of the 414 genes, and the neurons of the output layer correspond to the number of classes in the tested biological trait (softmax activation). Cross validation was used to estimate the performance of the classification models (10-fold; section “K-Fold Aross Validation”).

As the most basic neural network architecture we started by examining shallow neural networks with one non-linear hidden layer (ReLU activation), tested across an increasing number of hidden layer neurons (Figure 1A and Table 1, top). As expected, higher accuracy scores were obtained for shallow networks with higher numbers of hidden neurons in all three biological traits. Specifically, the classification models were found to be highly accurate in cases of extracellular stimulations and gender traits classification (98.69 and 95.9% mean accuracy scores, respectively). In contrast, predicting ethnicity fell shortly behind (mean accuracy of 76.19%). This concurs well with our general biological understanding that predicting stimulation trait would be highly accurate given that the genes within the input panel were chosen for their role in cellular response (Lee et al., 2014), that predicting gender would resolve in good performance given that gender-linked genes are included in the input panel, and that predicting ethnicity would obtain lower predictive scores as human population ethnicities are inherently mixed. We then tested two-hidden layers neural network architectures comprised of two non-linear hidden layers (ReLU activation; different number of hidden neurons in each layer; Figure 1B and Table 1, middle). Similar results were obtained for the two-layer architecture where increasing the number of neurons in all three cases improved the performance of the classifiers. However, the sole act of adding a second hidden layer did not seem to have an impact on the level of accuracy. Lastly, we have tested a multiclass logistic regression (softmax regression) through a simpler architecture, where no hidden layers (hence no ReLU activations) were introduced to the model. This architecture obtained a lower level of accuracy across all traits (Figure 1C and Table 1, bottom).

**TABLE 1:** Accuracy of multi-classification, obtained using the original gene expression levels.

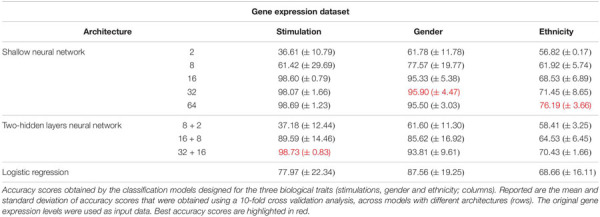

Next, we turned to examine the effect of data normalization on the performance of the neural network classification models. We applied standard Z-score normalization to the dataset across all samples to normalize the expression of each input gene. This pre-processing step is a common practice in gene expression analyses as distinct genes may vary by the shape of their expression distribution. We tested the model architectures described above on the normalized dataset and found that introducing gene normalization to the data strongly improved the accuracy of all classification models (Table 2). In particular, the two neural network architectures obtained outstanding performances even when lower numbers of neurons were used in the hidden layers (Table 2, top and middle). In the cases of extracellular stimulation and gender classification, the top accuracy scores were obtained for shallow networks with a much smaller number of neurons when normalized gene expression data was used compared to the non-normalized data (∼98% accuracy for stimulation trait using 8 versus 64 neurons for normalized versus non-normalized data, respectively, ∼96% accuracy for gender trait using 2 versus 32 neurons for normalized versus non-normalized data, respectively). The ethnicity classification model also showed dramatic improvement due to the normalization process: the accuracy is increased from 76.19% when applied on the original dataset to 90.75% when applied on the normalized dataset. Normalization also led to substantial improvement of logistic regression, which obtained similar (or even slightly better) accuracy scores compared to the neural network architectures (Table 2, bottom). We emphasize that this finding is important: as neural networks nowadays become a significant portion of the models investigated in computational biology, introducing complexity to the model does not necessarily improve model accuracy. When considering the tradeoff between model simplicity and model performance, special attention should be given to conventional models, such as logistic or linear regressions, which may be sufficient for various biological analyses. This conclusion is in agreement with previous studies (Montesinos-López et al., 2018; Traits et al., 2018).

**TABLE 2:** Accuracy of multi-classification, obtained using normalized gene expression levels.

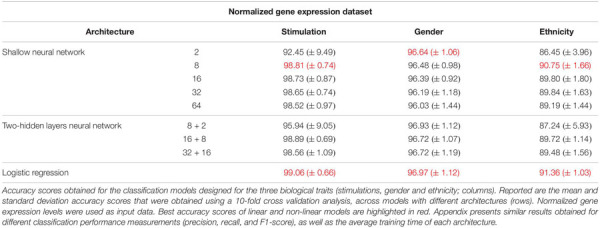

### Identifying the Genes That Are Important for Each Specific Class by Harnessing Deep Learning Visualization Techniques

Encouraged by the observation that neural networks can appropriately be used to classify biological traits based on molecular input data, we next aimed to identify the specific genes contributing to the classification model of each biological trait. For this purpose, we examined two commonly used deep learning visualization techniques – Saliency Maps and Activation Maximization.

**FIGURE 2:**
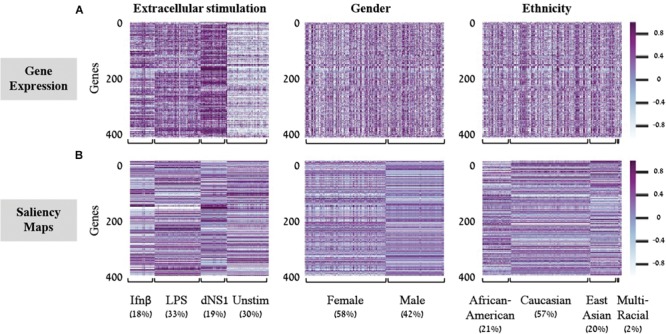
Gene expression versus saliency maps patterns. For each of the three biological traits (left to right panels) presented are heat maps of the gene expression data **(A)** or calculated saliency maps **(B)** across the 414 input genes (rows), for each sample in the test set (columns). Samples within each trait are grouped by their class label. Prominent patterns are mainly visible in the trained saliency maps model.

*Saliency Maps* are utilized to highlight the specific input patterns relevant for the process of assigning a sample to a particular class during model prediction (Simonyan et al., 2013; Kotikalapudi, 2017). For example, when a classifier is given an image of a bird, we might be interested to know whether it successfully classifies the image based on bird-related pixels or based on its surrounding leaves (Kotikalapudi, 2017). Formally, saliency maps quantify the saliency of each pixel – evaluating the change in the output caused by a small change in the tested input pixel (section “Saliency Maps and Averaged Saliency Maps”). In the context of our tabular numerical input we can utilize saliency maps to assess the contribution of each gene when a sample is assigned to a class by a trained classification model. In other words, the saliency map obtained for a given sample can be thought of as a numerical vector that quantifies the saliency – namely, the contribution – of each gene to the correct classification of the sample. Based on this rationale, we hypothesized that it would be possible to look for saliency maps patterns that are shared between all samples of a given class, and use these shared patterns as class-specific characteristic. To test this, we used shallow neural network architecture with 8 hidden neurons and the normalized gene expression dataset. Taking advantage of the tabular format of our structured data, the saliency maps can be presented as heatmaps, grouping samples according to their true class (Figure 2). Indeed, we find shared patterns of saliency maps for most samples within each class (Figure 2B). Furthermore, the substantial difference between traits suggests that the trained model is using a different subgroup of genes when testing the assignment of each class, as expected. These class-specific patterns were absent from the original (normalized) gene expression data (Figure 2A), emphasizing the utility of the model. Taken together, these observations support the notion that saliency maps patterns can be used to identify class-specific characteristics of biological traits.

**FIGURE 3:**
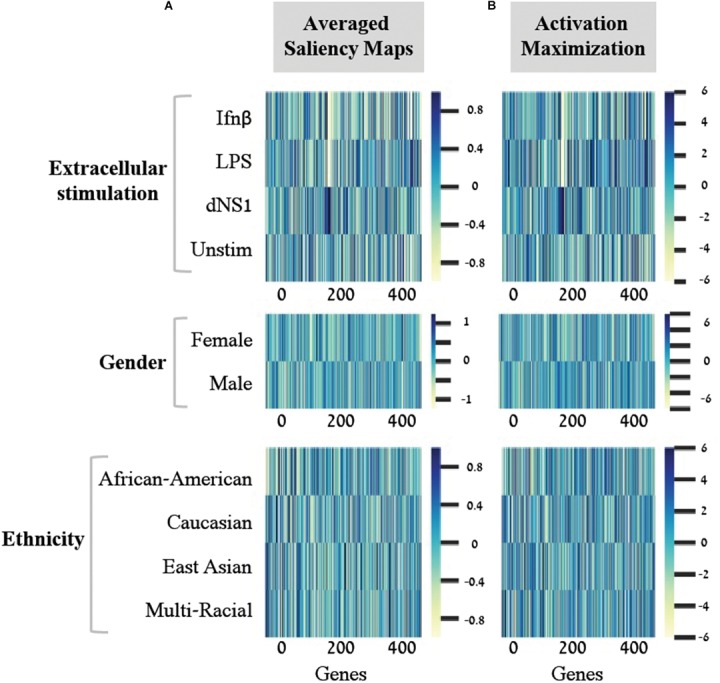
Scores for the importance of genes, with respect to each individual class. For each of the three biological traits (top to bottom) presented are heat maps of gene scores obtained for each class (*y*-axis) across the 414-genes input panel (*x*-axis). Gene scores were obtained using either the averaged saliency maps **(A)** or the activation maximization **(B)**. The comparison shows that both methods provide similar scores for the effect of genes on accurate prediction of each class.

In light of these findings, we set out to transform the saliency maps per sample in a model class into an aggregative saliency map per class in a biological trait. Our rationale is that if we consider the tabular structure of our data, we can calculate *Averaged Saliency Maps* for each class – where the saliency scores of a gene are averaged across all samples of a particular class (section “Saliency Maps and Averaged Saliency Maps”). More formally, we average the saliency scores of all samples in a certain class to form one saliency pattern that quantifies the contribution of each gene to the classification process of this class. Similarly, to the saliency maps per samples (Figure 2B), we can observe different patterns within each class in the averaged saliency maps as well (Figure 3A). Overall, the advantage of the tabular format of a structured input is exploited to highlight important genes of a class based on saliency scores. To the best of our knowledge, this is the first time that the tabular form is used for this purpose. Whereas the tabular structure is common in biological measurements, it is typically absent from classical deep learning research fields.

A second deep learning visualization technique, *activation maximization*, can also be used to uncover input patterns (genes) that are essential for classification. Activation maximization is commonly used to generate a synthetic input that best fits a trained model assignment to a particular class (Erhan et al., 2009; Chollet, 2015; Kotikalapudi, 2017). For example, when considering an image classification model, we might be interested in knowing how a sample that maximizes the activation of the birds’ class would look like. It might include a single bird within the image, but it might also encompass a bundle of beaks, wings and feathers (Chollet, 2015; Kotikalapudi, 2017). In our context, the activation maximization technique can be used to generate a gene pattern that maximizes the model assignment to a specific class within a biological trait. This will highlight the genes that have stronger influence on the prediction of a class, therefore pushing toward the assignment of a tested sample to its appropriate class. Formally, the activation maximization is calculated by generating an initial random input, followed by an iterative process of refining the generated input to maximize the neuron of interest (section “Activation Maximization”). Here, when testing one biological trait, we applied the activation maximization technique by generating a 414-genes input panel that maximizes the activation of the neuron that corresponds to the tested class. Figure 3 demonstrates comparison of scores between the averaged saliency maps (Figure 3A) and the activation maximization patterns (Figure 3B) on this data. The correlation between the scores calculated by the two methods is as high as 0.98 for each class within the three biological traits. Both methods are therefore found similarly, appropriate for assessing the contribution of a gene to the classification model, as expected. Taken together, our results support the notion that commonly used neural network visualization techniques can be used to pinpoint the genes that are important for the classification process, and further show that both methods provide similar scores when applied on structured data. To the best of our knowledge, this is the first time it was shown that both visualization techniques actually converge if an average is applied to the saliency maps.

### Identifying Genes That Are Generally Important for the Classification of a Biologic Trait

We next aimed to identify the genes that are most important to the classification of each biological trait. As the abovementioned gene importance scores refer to each biological class (Figures 2, 3), these scores should be aggregated across classes in order to indicate the general contribution of a gene to the classification of a trait. Such aggregation relies on the assumption that high-scored genes (with respect to particular classes) are also important for the entire classification process of the biological trait under study. We therefore average the absolute values of gene scores (obtained from one of the visualization techniques) across the classes. Absolute values are used since both highly positive and highly negative scores imply strong contribution to the correct classification of a class. We refer to these averaged scores as *trait-specific gene scores* and explore its use to prioritize genes that are important to each biological trait. The top K *trait-specific genes* are the K genes that obtained the highest trait-specific gene scores.

We first validated our basic assumption that the top trait-specific genes are most important for the classification process. To address this, we compared the accuracy obtained from a shallow neural network model whose input is a panel of all 414 genes to a network whose input is a subgroup panel of only top-K trait-specific genes. Given the high consistency of scores produced by the averaged saliency maps and activation maximization, results are shown only for the activation maximization method. We tested a variety of thresholds for the selection of trait-specific genes, and found that a small group of these genes may be sufficient for gaining similar performance to that of a 414-genes classifier, and that this is true for all three biological traits (Table 3, left), in agreement with our basic assumption.

**TABLE 3:** Accuracy scores obtained for classification based on the top trait-specific genes.

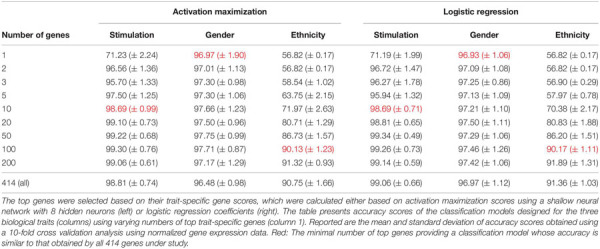

Regression coefficients are commonly used in biological studies as a method for evaluating the effect of each gene per model class. As logistic regression was previously shown to obtain comparable accuracy to that of a neural network, we reasoned that logistic regression could be used as a gold standard reference also for the assessment of gene scores. We therefore calculated trait-specific gene scores using conventional logistic regression, and subsequently compared these scores to those obtained using the visualization technique. In particular, we defined trait-specific gene scores (of logistic regression) as the average of absolute regression coefficients across all classes of a trait under study. We found that using the top K trait-specific genes for the classification of each trait, either selected based on the activation maximization or the logistic regression model, results in similar performance (Table 3). In addition, the K top trait-specific genes derived from both methods are quite similar (Table 4). These results suggest that visualization techniques-based prioritization may provide a good alternative to the conventional analysis that is commonly conducted through logistic regression, a fact that might become important in cases where deep neural networks may be required to solve more complex problems.

**TABLE 4:** Top ten trait-specific genes of each biological trait.

Stimulations		Gender		Ethnicity	

Activation maximization	Logistic Regression	Activation maximization	Logistic Regression	Activation maximization	Logistic Regression
IL6	IL6	ZFY	ZFY	PLA2G4C	PLA2G4C
IFIT2	IFIT2	EIF1AY	EIF1AY	GP1BA	GP1BA
PPP6C	IL7	DDX3Y	DDX3Y	LILRA3	IFITM3
IL1B	IL1B	DCBLD1	KCTD14	SPTLC2	CCL7
DTX4	IFI44	TLR8	C12orf23	OTOF	SLC8A1
IL28A	DTX4	CTSH	LILRA3	MSR1	ATP1B1
IFI44	IRG1	IL1F9	NRIP3	IFITM3	CD40
IL28B	SCG3	GPR68	TLR8	LHFP	CTSH
IL29	IL1RN	MGC57346	HPS1	CUL4A	SIGLEC9
PTX3	IL28A	ERAP1	IL4R	SLC8A1	C6orf192

*For each trait (top row), presented are ten genes that obtained the top (highest) trait-specific gene scores, calculated using either activation maximization (left) or logistic regression (right).*

Finally, we investigate the biological role of the top prioritized genes predicted for each trait. We define the *leading trait-specific genes* as the minimal set of K genes that allow accuracy that is comparable to that of the full set of genes. For instance, in the case of classifying the extracellular pathogenic stimulation, 10 top genes is the minimal set whose accuracy is similar to the accuracy obtained by 414 genes (>98%, Table 3, left; row 5 versus 10); these 10 genes are therefore the leading stimulation-specific genes. These leading stimulation-specific genes (Table 4; left) are found to be primarily immune-cells mediated cytokines (IL6, IL28A, IL28B, IL29, IL1B) and immune-defense genes (IFIT2, IFI44), which serve as the innate immune defense line against invading pathogens (Iwasaki and Medzhitov, 2004). In fact, the top two genes – IL6 and IFIT2 – are solely sufficient for the stimulation-trait classifier and gain excellent accuracy (above 96%) when they are both used as the input data (Table 3, second row). In the case of gender classification, a single top gene was found as the leading gene with exceptional performance (>96%, Table 3, middle). In fact, each of the top three genes – ZFY, EIF1AY or DDX3Y – can serve as the sole input of a gender classification model with >96% accuracy (data not shown). This is perhaps unsurprising, in retrospect, given that all these three genes are Y-linked genes (Vakilian et al., 2015). Finally, in the more inherently complex biological trait – ethnicity – a much larger group of about 100 leading genes is needed in order to preserve sufficient accuracy of around 90%.

To summarize this work, we propose a thorough examination of utilizing neural networks to classify biological traits and demonstrate the use of two classical deep learning visualization techniques – saliency maps and activation maximization – to highlight input patterns essential for the classification model of each trait. We demonstrate how to reveal the most important genes for each classification – which we term leading trait-specific genes – and show strong biological reasoning for why these genes were selected by the model to guide the prediction process. As opposed to inference conducted through regression coefficients, the proposed use of visualization techniques on structured data to examine feature importance can be effortlessly extended to deep neural network architectures that might be found crucial for classifying various complex traits. As the application of neural networks in computational biology is a rapidly growing field, we believe these techniques provide a powerful and general approach for identifying the particular input features essential for the prediction of a trained neural network model.

## Materials and Methods

### Gene Expression Data and the Classified Biological Traits

We utilized a published dataset (Lee et al., 2014) (GEO accession GSE53166) consisting of the expression levels of 414 immune-related genes in peripheral blood monocyte-derived dendritic cells (DC) extracted from healthy individuals. In total, 2441 samples are included in the dataset – each sample is treated in our analysis as a 414-genes expression panel. Each sample is accompanied with information regarding the gender and the ethnicity of the individual from which the immune cells were derived and information regarding an extracellular stimulation following which the genes expression levels were measured (or indicated that it was measured without prior extracellular stimulation). The distribution of the 2441 samples across the different classes within the three biological traits – gender, ethnicity, and extracellular stimulation – is summarized in Table 5. Normalization of the gene expression data was conducted using *Z*-score normalization applied on each gene across all 2441 samples.

**TABLE 5:** Biological traits.

Biological trait	Classes
Extracellular stimulation	Unstim (734 samples), LPS (806 samples), dNS1 (469 samples), IFNβ (432 samples)
Gender	Female (1412 samples), Male (1029 samples)
Ethnicity	African-American (506 samples), Caucasian (1387 samples), East-Asian (487 samples), Multi-racial (61 samples)

*The three biological traits examined in this study (left), presented together with their corresponding classes and the number of samples included in each class (right). Abbreviations of stimulations: Unstim, unstimulated sample; LPS, lipopolysaccharide; dNS1, influenza virus lacking the NS1 viral gene; IFNβ, the interferon-β cytokine.*

The selection of this dataset, which is a relatively small one, serves two goals: first, we aimed to use a dataset for which conventional methods provide high performance (thereby allowing systematical comparison of feature importance), and second, we aimed to highlight the fact that for many datasets, conventional methods may be sufficient for the construction of an accurate classifier.

### Neural Network Architectures

We tested two different neural network architectures for the multiclass classification problem for each of the three biological traits – a neural network with a single hidden layer (here termed “shallow neural network”; Figure 1A) and a neural network with two hidden layers (“two hidden layers neural network”; Figure 1B). Different dimensions were tested for the hidden layers (2, 8, 16, 32, or 64 for the shallow network; 8+2, 16+8, or 32+16 for the two hidden layers network). ReLU activation function was used and a standard L2 regularization with λ = 0.01 was applied (no hyperparameter tuning was conducted for choosing λ). The input for all classifiers is the expression panel of the 414 immune-related genes, while the number of output neurons corresponds to the number of classes in each trait (2 for gender, 4 for ethnicity and extracellular stimulation). A softmax activation function was used at the output layer to assign a class for a tested sample. Lastly, we formed a neural network-like instance of logistic regression to preserve consistency with the abovementioned neural networks. The logistic regression network was constructed as an input layer directly connected to an output layer with softmax activation (Figure 1C; no hidden layer).

### K-Fold Aross Validation

In order to properly estimate and compare the performance of the different neural network classification models, we used 10-fold cross validation strategy to split the dataset multiple times (Kohavi, 1995; Dietterich, 1998). Each split produced a training set, on which a neural network was trained, along with a test set, which was used to evaluate the accuracy of the trained model. We used stratified cross validation to preserve the proportion of the samples within each class in each split. The results of the 10-fold cross validation splits are summarized as the mean and standard deviation scores, providing a robust method for performance assessment.

### Saliency Maps and Averaged Saliency Maps

Saliency Maps is a deep learning visualization technique commonly used for highlighting the input components within a sample that are important for the process of assigning the sample to its particular class by a trained neural network model (Simonyan et al., 2013). Formally, this method measures the contribution of each input component to the classification process. An input component can be, for example, a pixel within an image (in image classification) or a gene within an expression panel (in classification of biological samples). In other words, saliency maps quantify the impact that small changes in the input data have on the correct classification of each sample (Kotikalapudi, 2017).

In this study we use saliency maps as a tool for exploring the contribution of each gene to the correct classification of a sample within a biological trait. The saliency maps formally provide a score for each gene per sample. We further exploit the tabular structure of gene expression data to create “Averaged Saliency Maps,” where an average score is calculated for each class across all its samples. Such averaged maps emphasize the genes that are important for the assignment of each class, taking into consideration the scores of all samples in these classes. Importantly, such analysis is not possible in the general case of image classification (which relies on pixels that do not necessarily share the same topological structure). Here, the averaging is only possible due to the tabular organization of the molecular data across multiple samples. We use this concept of averaged saliency maps to move from per-sample characteristics to class-specific characteristics.

### Activation Maximization

Activation Maximization is a deep learning visualization technique that is commonly used for generating an input instance that maximizes the activation of a particular filter within a trained model (Erhan et al., 2009; Chollet, 2015). Specifically, activation maximization can be used to generate an input that maximizes the activation of an output neuron corresponding to a particular class. Formally, activation maximization process is conducted by generating an initial random input and iteratively refining it to maximize a particular class (Kotikalapudi, 2017). In this study we use the activation maximization to evaluate the contribution of each of the genes to the process of assigning samples to a particular class (within a biological trait under study). High activation maximization gene scores (either positive or negative) indicate a greater impact of a gene on the classification process of the tested class.

### Trait-Specific Genes

Given the contribution scores of each gene per class (either based on activation maximization or on averaged saliency maps), averaging these scores across the classes allows the detection of genes that are important for the classification of the entire biological trait. We refer to such averaged scores as “trait-specific gene scores,” and top-ranked genes based on these scores are referred to as “trait-specific genes.” Finally, the “leading trait-specific gene” are the minimal group of trait-specific genes whose classification accuracy is similar to that obtained by the full input set of genes.

## Discussion

During the past decade, neural networks have emerged as a promising tool widely used in complex classification analysis, standing at the frontline of various deep learning fields (Lecun et al., 2015). Recent studies in biomedicine have naturally proposed utilizing gene expression data in order to classify medical traits through neural networks (Chen et al., 2014; Dwivedi, 2018; Eraslan et al., 2019), a trend that can only be expected to continue to thrive in the coming years. In this study we examine the classification of different biological traits based on gene expression levels derived from healthy individuals, focusing on two main contributions: first, we present a methodological approach to address basic questions revolving the use of neural networks. We discuss the selection of an appropriate architecture while considering the tradeoff between model complexity and accuracy. Second, we propose the use of two common deep learning visualization techniques to explore genes contribution, per sample or per trait class, to the classification process. We show how these methods can be used to uncover genes that are essential for the classification process of a given biological trait.

We focused on three biological traits – an extracellular pathogenic-like stimulation following which gene expression levels were measured, the gender of the person from which a sample was derived and the person’s ethnicity. We started by exploring different neural network architectures for the prediction of each of the three biological traits and found that in all three cases, simple architectures were sufficient to obtain highly accurate predictions. We further demonstrated that data normalization greatly improves the performance of the network models, enabling the use of smaller, hence more efficient, neural networks. We also found that when using the normalized dataset, a simple logistic regression obtained accuracy scores that are similar to those obtained by neural network models. The tradeoff between model simplicity versus model accuracy should be therefore taken into consideration before turning to the use of a more complex neural network model.

Next, we explored the use of two deep learning visualization techniques – Saliency Maps (Simonyan et al., 2013; Kotikalapudi, 2017) and Activation Maximization (Erhan et al., 2009; Chollet, 2015) – for the purpose of revealing trait-specific genes essential for the classification model trained for each biological trait. We used the two methods to investigate input patterns (in our case, genes) that are important for the classification process of a particular trait. We have leveraged the tabular form of the gene expression data to show that the two visualization techniques converge to similar results. As expected, the prioritization of the genes based on their contribution scores resulted in a different group of leading trait-specific genes suggested for each of the classified traits, including Y-linked genes for gender classification and immune-cells mediated cytokines for extracellular stimulation classifier. We took advantage of the fact that the logistic regression models were found comparable to the neural network models in order to show high concurrence between the essential genes proposed by the two visualization techniques with those having the largest (absolute) coefficients in a logistic regression model. Taken together, these findings support the notion that deep learning visualization techniques can be used as valid methods for exploring the importance of omics components in various biomedical fields. Furthermore, our results lay strong foundations for the general utility of visualization techniques for interpretability in the context of any complex structured-input neural networks.

## Data Availability Statement

Data was downloaded from GEO, accession number GSE53166.

## Code Availability

The full analysis – including code implementation of the saliency maps and activation maximization methods as well as the datasets used – is available on https://github.com/roniwile/neural-network-visualization-methods-for-gene-expression-data.

## Author Contributions

Both authors listed have made a substantial, direct and intellectual contribution to the work, and approved it for publication.

## Conflict of Interest

The authors declare that the research was conducted in the absence of any commercial or financial relationships that could be construed as a potential conflict of interest.

## Appendix

Performance analysis of multi-classification models, obtained using normalized gene expression levels. Performance scores (accuracy, precision, recall, F1-score; columns) obtained for the classification models designed for the three biological traits (stimulations, gender and ethnicity; top to bottom) along with training time (in seconds). Reported are the mean and standard deviation scores that were obtained using a 10-fold cross validation analysis, across models with different architectures (rows). Normalized gene expression levels were used as input data.

**Table:**

Stimulation						
Architecture		Accuracy	Precision	Recall	F1-score	Time (sec)
Shallow neural network	2	92.46 (± 10.12)	92.10 (± 14.02)	92.46 (± 10.12)	90.93 (± 13.27)	4.60
	8	98.81 (± 0.83)	98.82 (± 0.83)	98.81 (± 0.83)	98.81 (± 0.83)	6.04
	16	98.77 (± 1.11)	98.81 (± 1.05)	98.77 (± 1.11)	98.77 (± 1.11)	7.00
	32	97.54 (± 1.69)	97.74 (± 1.42)	97.54 (± 1.69)	97.54 (± 1.69)	7.24
	64	98.52 (± 1.01)	98.57 (± 0.95)	98.52 (± 1.01)	98.52 (± 1.02)	12.04

Two-hidden layers neural network	8 + 2	98.44 (± 0.80)	98.47 (± 0.77)	98.44 (± 0.80)	98.44 (± 0.80)	9.54
	16 + 8	98.44 (± 1.38)	98.52 (± 1.27)	98.44 (± 1.38)	98.44 (± 1.38)	10.93
	32 + 16	98.61 (± 0.97)	98.65 (± 0.90)	98.61 (± 0.97)	98.60 (± 0.98)	12.85

Logistic regression		98.81 (± 0.76)	98.84 (± 0.73)	98.81 (± 0.76)	98.81 (± 0.76)	11.59

**Table:**

Gender						
Architecture		Accuracy	Precision	Recall	F1-score	Time (sec)
Shallow neural network	2	96.56 (± 1.06)	96.60 (± 1.04)	96.56 (± 1.06)	96.56 (± 1.06)	3.99
	8	96.39 (± 1.83)	96.41 (± 1.83)	96.39 (± 1.83)	96.39 (± 1.84)	4.98
	16	96.52 (± 1.37)	96.55 (± 1.36)	96.52 (± 1.37)	96.52 (± 1.37)	6.06
	32	96.72 (± 1.77)	96.76 (± 1.74)	96.72 (± 1.77)	96.72 (± 1.77)	7.16
	64	95.62 (± 1.99)	95.70 (± 1.90)	95.62 (± 1.99)	95.61 (± 2.00)	9.51

Two-hidden layers neural network	8 + 2	96.89 (± 1.62)	96.93 (± 1.62)	96.89 (± 1.62)	96.89 (± 1.62)	9.67
	16 + 8	96.52 (± 1.18)	96.55 (± 1.18)	96.52 (± 1.18)	96.52 (± 1.18)	10.92
	32 + 16	96.43 (± 1.52)	96.50 (± 1.42)	96.43 (± 1.52)	96.44 (± 1.51)	12.84

Logistic regression		96.93 (± 0.97)	96.98 (± 0.94)	96.93 (± 0.97)	96.93 (± 0.97)	14.62

**Table:**

Ethnicity						
Architecture		Accuracy	Precision	Recall	F1-score	Time (sec)
Shallow neural network	2	85.71 (± 4.22)	83.84 (± 4.09)	85.71 (± 4.22)	84.22 (± 4.61)	5.04
	8	90.17 (± 1.80)	88.37 (± 1.88)	90.17 (± 1.80)	89.18 (± 1.81)	5.05
	16	90.38 (± 1.56)	88.76 (± 2.08)	90.38 (± 1.56)	89.36 (± 1.67)	5.88
	32	88.40 (± 3.22)	86.96 (± 3.31)	88.40 (± 3.22)	87.49 (± 3.21)	8.66
	64	86.63 (± 3.92)	85.07 (± 3.61)	86.63 (± 3.92)	85.69 (± 3.76)	11.29

Two-hidden layers neural network	8 + 2	88.00 (± 3.80)	86.01 (± 3.61)	88.00 (± 3.80)	86.78 (± 3.91)	9.74
	16 + 8	89.80 (± 2.24)	87.79 (± 2.01)	89.80 (± 2.24)	88.66 (± 2.18)	11.99
	32 + 16	89.03 (± 2.70)	87.08 (± 2.36)	89.03 (± 2.70)	87.92 (± 2.61)	13.35

Logistic regression		91.48 (± 0.86)	90.13 (± 1.15)	91.48 (± 0.86)	90.62 (± 0.94)	11.57
